# Screening Cases of Suspected Early Stage Chronic Kidney Disease from Clinical Laboratory Data: The Comparison between Urine Conductivity and Urine Protein

**DOI:** 10.3390/biomedicines11020379

**Published:** 2023-01-27

**Authors:** Ming-Feng Wu, Ching-Hsiao Lee, Po-Hsin Pai, Jiunn-Min Wang

**Affiliations:** 1 Department of Internal Medicine, Division of Chest Medicine, Taichung Veterans General Hospital, Taichung 407, Taiwan; 2 Department of Medical Laboratory Science and Biotechnology, Central Taiwan University of Science and Technology, Taichung 406, Taiwan; 3 Department of Medical Technology of Medicine, Nursing and Management, Miaoli 350, Taiwan; 4 Department of Pathology & Laboratory Medicine, Taichung Veterans General Hospital, Taichung 407, Taiwan

**Keywords:** chronic kidney disease (CKD), estimated glomerular filtration rate (eGFR), urine conductivity, sensitivity, urine protein

## Abstract

(1) Background: Chronic kidney disease (CKD) affects more than 800 million global population. Early detection followed by clinical management is among the best approaches for the affected individuals. However, a sensitive screening tool is not yet available. (2) Methods: We retrospectively reviewed 600 patients aged >20 years with a full range of estimated glomerular filtration rate (eGFR) for clinical assessment of kidney function between 1 January 2020, to 30 April 2021, at the Taichung Veterans General Hospital, Taichung, Taiwan. With stratified sampling based on the level of eGFR, participants were evenly grouped into training and validation sets for predictive modeling. Concurrent records of laboratory data from urine samples were used as inputs to the model. (3) Results: The predictive model proposed two formulae based on urine conductivity for detecting suspected early-stage CKD. One formula, P_male45, was for used male subjects aged ≥45 years, and it had a prediction accuracy of 76.3% and a sensitivity of 97.3%. The other formula, P_female55, was used for female subjects aged ≥55 years. It had a prediction accuracy of 81.9% and a sensitivity of 98.4%. Urine conductivity, however, had low associations with urine glucose and urine protein levels. (4) Conclusion: The two predictive models were low-cost and provided rapid detection. Compared to urine protein, these models had a better screening performance for suspected early-stage CKD. It may also be applied for monitoring CKD in patients with progressing diabetes mellitus.

## 1. Introduction

Kidneys are responsible for maintaining the homeostasis of body fluids by regulating the balance of water, electrolytes, and acid-base [1,2]. It is achieved through filtration at the glomeruli, secretion, and reabsorption in the tubular region. For instance, the tubular cells reabsorb more bicarbonate from the urine, the kidneys secrete more hydrogen ions into urine, and ammonia genesis leads to an increase in the formation of the NH3 buffer in response to acidosis [3]. In the same manner, body components, such as albumin or glucose, are sufficiently retained in the blood, with excess amounts excreted in the urine [3,4]. The kidney also transports toxins from the blood into the urine with active processes. [1,5]. These toxins include small-size water-soluble compounds (e.g., creatinine and urea), medium-size compounds (e.g., cystatin-C and β2-microglobulin) and protein-bound uremic toxins (PBUTs; e.g., indoles and phenols) [5]. The kidney can also secrete various hormones and humoral factors like hormones in the renin-angiotensin system (RAS), erythropoietin (EPO), and 1.25 dihydroxy vitamin D3 [6]. When the kidneys are injured, the result is poor functioning for homeostasis, filtration, and hormone regulation. A number of comorbidities will appear. They include hypertension and anemia [7].

Chronic kidney disease (CKD) is defined as abnormalities of kidney structure or function for over three months according to the guidelines of KDIGO (Kidney Disease: Improving Global Outcomes) 2021 [8]. Based on the estimated glomerular filtration rate (eGFR), serum creatinine, age, race, sex, and body size, CKD is categorized as stage 1 (G1): with normal eGFR (mL/min/1.73 m^2^) of ≥90; stage 2 (G2): with mildly decreased eGFR of 60–89; stage 3 (G3): with moderated decreased eGFR of 30–59; stage 4 (G4): with severely decreased eGFR of 15–29, and stage 5 (G5): with eGFR < 15 and kidney failure. CKD affects >10% of the general population, or >800 million global population [9]. CKD is also age- and gender-dependent [9,10]. A previous report in 2021 indicated that CKD affects 12% of those aged 45–64 years and up to 38% of those over 65 years in the United States [10]. In the same report, CKD occurs slightly more in women than in men (14% vs. 12%).

CKD poses a significant risk for cardiovascular morbidity and mortality [11]. It is also associated with diabetes mellitus (DM), obesity, blood pressure, etc., resulting in poor life quality [12,13]. Hence, the medical cost of treating the late stages of CKD is high. Early detection, followed by appropriate clinical management, is desirable for caring for affected individuals [14,15]. Since filtration at glomeruli is impaired during the initial stage of CKD, protein is excreted into the urine. Therefore, urine protein (UP) is a marker for mortality in CKD [16,17]. Urine dipsticks represent an inexpensive, accessible, and widely used test for proteinuria screening. The test is the reference of albumin-creatinine ratio (ACR) on a random spot urine sample [18,19]. The dipstick result has 69.4% sensitivity and 86.8% specificity for trace or ACR ≥ 30 mg/g [19]. A population-based study showed that for the same level of ACR, the urine dipstick test has a poor performance in detection at a sensitivity of 43.6% [20]. A systematic review and meta-analysis concluded that the dipstick might interfere with ketone, glucose, or antibiotics in the urine, resulting in false negative findings [21]. An alternative, simpler, low-cost, and more sensitive method to detect early CKD is in demand.

The conductivity of an electrolyte solution, like urine, is a simple, cheap, and fast parameter to assess its electrical impedance [22]. A previous study indicated that urine conductivity (UCond), correlated with its creatinine concentration, is a potential marker for renal dysfunction [23]. Our previous study found that UCond provides a better screening ability than UP for subjects with early-stage CKD (i.e., those with eGFR ≥ 60 mL/min/1.73 m^2^) [24]. Based on such a finding, we supposed that the inclusion of conductivity as an input variable would have the potential to be developed as a prediction module for screening suspected early-stage CKD.

## 2. Materials and Methods

### 2.1. Study Design, Setting, and Population

**Figure 1:**
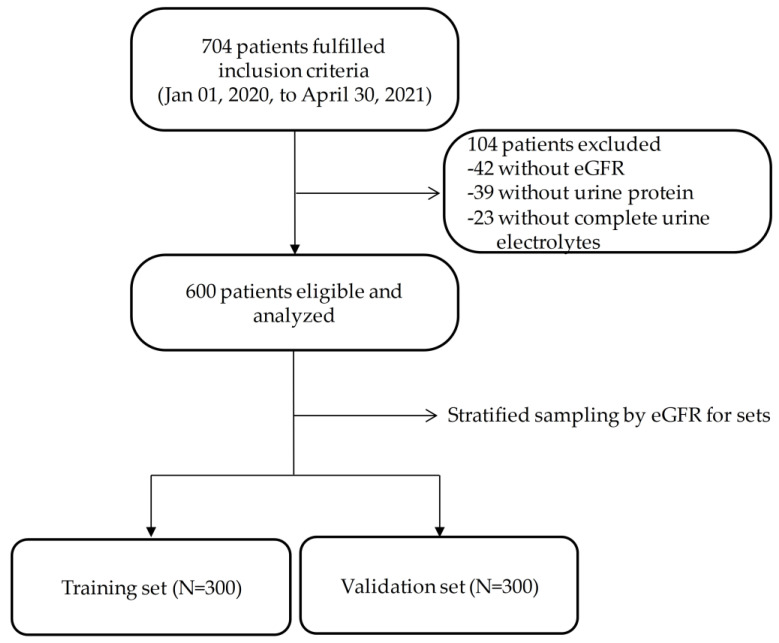
The patient enrollment flow chart.

We retrospectively reviewed patients aged > 20 years with a full range of eGFR for clinical assessment of kidney function between 1 January 2020, to 30 April 2021, received at the Taichung Veterans General Hospital, Taichung, Taiwan. In total, 704 patients fulfilled the inclusion criteria and enrolled initially. In total, 104 patients were excluded since they were without eGFR, urine protein, or complete urine electrolytes (Figure 1). A total of 600 patients were enrolled in the final analysis. Laboratory data on urine samples taken at the same time were analyzed. 

The Institutional Review Board and Ethics Committee of Taichung Veterans General Hospital approved our study (approval number: CE21164B). Informed consent was waived due to its retrospective and electronic medical chart review nature.

### 2.2. Estimated Glomerular Filtration Rate

The estimated glomerular filtration rate (eGFR) was reported by the guidelines of KDIGO [7] with the following Formula (1):

(1) $$GFR\left(\mathrm{mL}/\min/1.73\;m^{2}\right)=175\times {\left(Scr\right)}^{-1.154}\times {\left(Age\right)}^{-0.203}\times \left(0.742\;if\;female\right)$$

where *Scr* (mg/dL) is the concentration of serum creatinine, it was determined with the enzymatic method provided by LABOSPECT 008 AS (Hitachi High-Tech Co., Ibaraki, Japan). The suspected early-stage CKD (stage 1 and stage 2) was defined as those participants with an eGFR ≥ 60 (mL/min/1.73 m^2^).

### 2.3. Laboratory Data with a Urine Sample

#### 2.3.1. Urine Quantitative Analysis

Urine protein (UP), urine glucose (UG), and urine creatinine (UC) levels were determined, respectively, based on the Turbidimetric, Hexokinase method, and enzymatic methods, using the LABOSPECT 008 AS (Hitachi High-Tech Co., Ibaraki, Japan). The normal range was set at <15 mg/dL, while the positive range was set at ≥15 mg/dL.

#### 2.3.2. Urine Electrolytes

Urine Na^+^, urine K^+^, urine Cl^−^, and urine Ca^++^ were routine measurements of urine electrolytes. They can be determined with the ION SELECTIVE ELECTRODE method by LABOSPECT 008 AS (Hitachi High-Tech Co., Ibaraki, Japan).

#### 2.3.3. Urine Conductivity and Osmolality

Urine conductivity represents the ability of urine to conduct electric current. It is the reciprocal of the resistance measured when the current passes through a liquid column with a length of 1 cm and a cross-sectional area of 1 cm^2^. Urine osmolality measures the number of dissolved particles per unit of water in the urine. Both urine conductivity and osmolality were measured with Sysmex UF-5000 (SYSMEX CORPORATION, Kobe, Japan).

#### 2.3.4. Urine Specific Gravity

Specific gravity (S.G., mass of a unit volume) is the density ratio of urine with respect to water. It was measured with Sysmex UF-5000 (SYSMEX CORPORATION, Kobe, Japan).

### 2.4. Statistical Analyses

#### 2.4.1. Sets Grouping

With stratified sampling data based on their level of eGFR, participants were evenly grouped into training and validation datasets for later predictive modeling. Predictive formulae based on urine conductivity (UCond) were developed in the training set. The predicted values were tested using the unseen validation dataset. 

#### 2.4.2. Predictive Models Development

Since eGFR was estimated with age, sex, and serum creatinine, age is a potential variable of the predictive models. Therefore, age was merged with UCond to create a stepwise regression model once it had shown a moderate correlation (*r* ≥ 0.3) with eGFR in the training set. To identify other potential variables that may interfere with UCond, their correlations between laboratory data with a urine sample were analyzed.

#### 2.4.3. The Better Fitness Population for the Predicted Model

Since age was a non-linear component for eGFR, the proposed regression model may cause more bias for some age populations. To strengthen the quantification of screening the suspected cases of early-stage CKD, the area under the curve (AUC) > 0.7 of UCond against age was set as the threshold of fitness for the prediction model.

#### 2.4.4. The Validation and the Comparison

Predicted errors were assessed with a Bland–Altman plot on the validation set [25]. Since UP levels are a sensitive marker of those cases of CKD covering early to advanced stages [17], the accuracies of both UP (<15 mg/dL) and the prediction model were determined. The true positive screening was set in the condition with eGFR ≥ 60 (mL/min/1.73 m^2^). The sensitivity (Sn), specificity (Sp), positive predictive value (PPV), and negative predictive value (NPV) were performed [26]. The continuous variables of urine and blood data were expressed as mean and standard deviation (SD). Categorical variables were represented by numbers (percentages). Statistical analyses were performed using the SPSS software version 18.0 (SPSS Inc., Chicago, IL, USA) with statistical significance set at *p* < 0.05.

## 3. Results

A total of 600 patients, 61.9 ± 15.0 years old, with 332 males (55.3%) and 268 females (44.7%), were enrolled in the final analysis (Table 1). Their eGFR was 47.7 ± 26.6 (mL/min/1.73m^2^). The number (and percentage) of patients in the five groups of eGFR values were 39 (6.5%) ≥ 90, 136 (22.7%) for 60–89, 268 (44.7%) for 35–59, 84 (14.0%) for 15–29, and 73 (12.2%) for < 15. Their UCond was 10.8 ± 4.6 (mEq/L), and urine protein (UP) was 81.3 ± 192.3 (mg/dL). For those who tested negative for UP (<15 mg/dL), UP was 7.5 ± 3.2 (mg/dL). Other laboratory data are shown in Table 1. There was no significant difference between the training and validation datasets.

Serum creatinine for males was 2.24 ± 1.78 (mg/dL). It was higher than females at 2.01 ± 2.11 (mg/dL). However, there was no significant difference between males and females (Table 2). Neither the age nor eGFR was so. In addition, urine specific gravity, urine glucose, and urine creatinine were significantly higher for males than for females.

The value of eGFR with which the analysis of Pearson correlation coefficients between UCond and eGFR in the training set was performed (Table 3). Results showed that Pearson correlation coefficients between UCond and eGFR in the training set were 0.350 for males and 0.385 for females. Age and eGFR showed a significant correlation coefficient of −0.385 for females and −0.170 for males. Therefore, age was a candidate variable for training the prediction model only in the case of females. It was noted that eGFR was significantly correlated to urine protein at −0.419 but without a significant correlation to urine glucose of 0.416. This non-statistical significance may be due to the fact that the number of urine glucose samples was only 53 in males (Table 2). On the other hand, UCond was highly correlated with urine electrolytes. For all urine samples in the training set, the correlation coefficient varied from 0.517 to 0.893 for urine Ca^++^ to Cl^−^, respectively. Moreover, we found a correlation coefficient of 0.628 between UCond and urine S.G. and 1.000 between UCond and urine osmolality. We also noted the very low correlation coefficients for UCond between urine protein, urine glucose, and age.

To evaluate the performance of the proposed prediction with different UCond for specific ages, the AUC results showed a value of 0.723 for females aged ≥55 years and 0.703 for females aged ≥50 and with eGFR of 60.0 (mL/min/1.73 m^2^); while AUC was 0.712 for males aged ≥50 and 0.710 for males aged ≥45 with eGFR of 60.0 (mL/min/1.73 m^2^) (Table 4). However, there was no age applicable with AUC over 0.7 with eGFR at 30.0 (mL/min/1.73 m^2^). By the age-applicable standard, a total of four predictive models, including UCond and/or age for eGFR, were proposed (Table 5). For males aged ≥45, the predicted eGFR was

(2) 25.541+1.847×UCond

For males aged ≥50, the predicted eGFR was

(3) 26.686+1.768×UCond

For females aged ≥50, the predicted eGFR was

(4) 69.563+2.303×UCond–0.752Xage

For females aged ≥55, the predicted eGFR was

(5) 78.160+2.217×UCond–0.854×age

**Figure 2:**
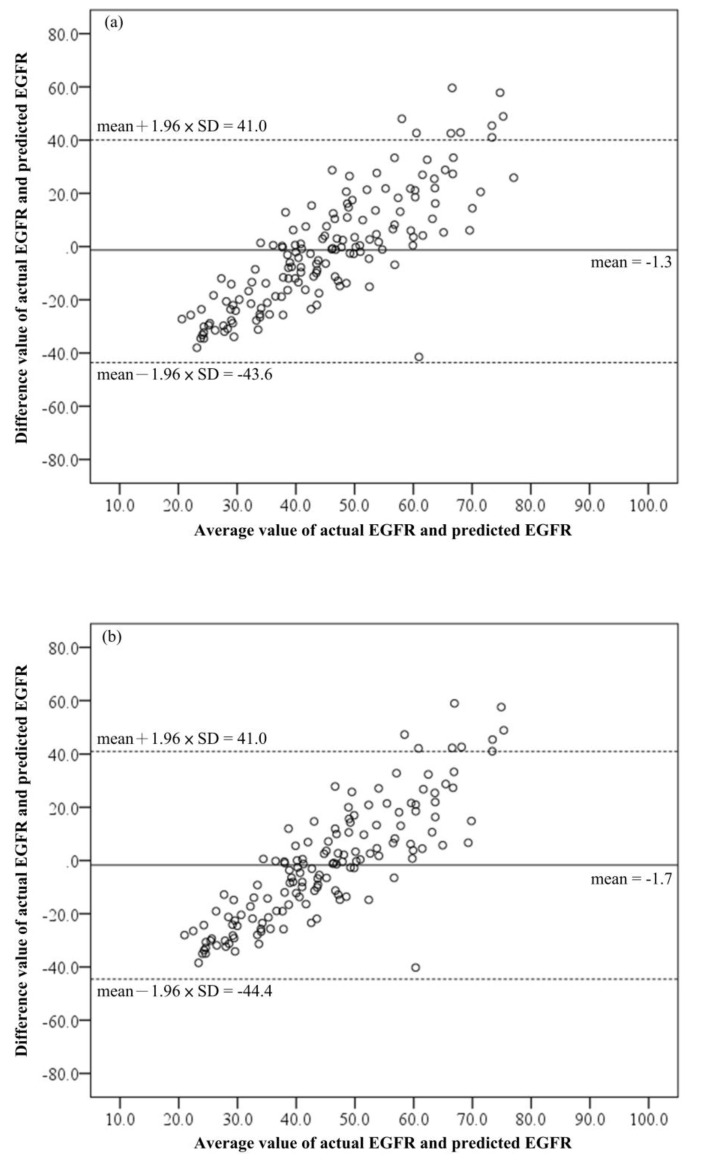

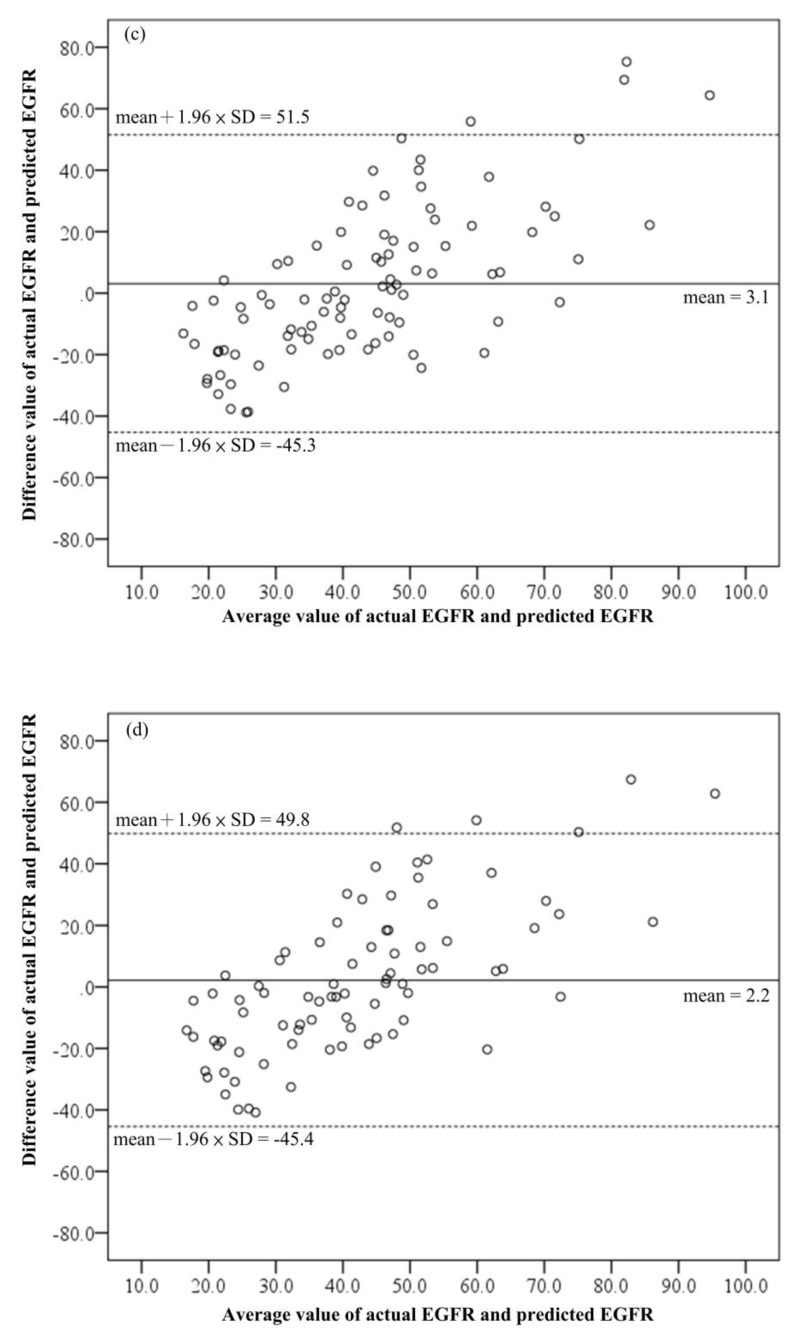
Bland–Altman plot between predictions and actual value. (**a**) Actual eGFR versus predicted eGFR for males aged ≥45; (**b**) Actual eGFR versus predicted eGFR for males aged ≥50; (**c**) Actual eGFR versus predicted eGFR for females aged ≥50; (**d**) Actual eGFR versus predicted eGFR for females aged ≥55.

The four predictive models gave prediction errors of −1.3 ± 21.6, −1.7 ± 21.8, 3.1 ± 24.7, and 2.2 ± 24.3, respectively, using the above formulae from (2) to (5) with the validation set (Table 5). In addition, they displayed a right tilt upward on the Bland–Altman plot (Figure 2). This feature indicated that the higher the eGFR, the higher the predicted errors. For screening the suspected early-stage CKD (eGRF ≥ 60) for males aged ≥45 with Formula (2), the sensitivity (Sn) was 97.3%, and accuracy (Acc) was 76.3%. The performance was much better than that using the UP values (Sn: 67.5%, Acc: 63.8%). Formula (2) was renamed ‘P_male45’ for clarity. For females aged ≥55 with Formula (5), the Sn was 98.4%, and Acc was 81.9% (Table 6). This performance was similarly much better than that using the UP (Sn: 75.0%, Acc: 61.4%). We also renamed Formula (5) ‘P_female55’. Compared with UP, the two predictive models presented a lower specificity (Sp) (17.5% and 30.0%, respectively), and their negative predictive value (NPV) was 70.0% and 85.7%, respectively, which was lower than UP (which was only 84.3% and 87.8%, respectively). For enrolled male subjects aged ≥ 50, Sn, Sp, Acc, PPV and NPV were 99.1%, 7.9%, 75.2%, 75.2%, and 75.0%, respectively; and for females, 94.2%, 20.8%, 75.3%, 77.4%, and 55.6%, respectively. Although they presented the highest Sn for suspected early-stage CKD screening in both genders, their lowest Sp caused many false positives. Overall, both P_male45 and P_female55 could therefore be more useful in clinical practice even though the study enrolled patients aged more than 20 years old.

## 4. Discussion

We have proposed four predictive models to detect patients with early-stage chronic kidney disease (CKD). Among these models, P_male45 combined with urine conductivity (UCond) for male patients aged ≥45 gave an accuracy of 76.3%, while P_female55 with UCond and age gave an accuracy of 81.9% for females aged ≥55. These results were all better than those based on urine protein (63.8% accuracy for males aged ≥45 and 61.4% accuracy for females aged ≥55). Our results also showed that urine protein and urine glucose were poorly correlated with UCond. The UCond-based predictive models for eGFR may be less influenced by proteins present in the urine of patients with an injured kidney or glucose present in the urine of patients with progressive diabetes mellitus. This result is consistent with those previously reported [23,27].

The kidney plays a critical role in the control of electrolyte balance. In response to dietary intake and endogenous production or drug intake, the excretion of electrolytes into urine is regulated by the kidney [28]. Accordingly, urine electrolytes are widely used in the diagnostic interpretation of hypovolemia, kidney injury, and acid-base and electrolyte disturbances [29,30].

Levels of sodium (Na^+^), potassium (K^+^), chloride (Cl^−^), and calcium (Ca^++^) are used for routine assessments in urine analysis. Through co-transportation with glucose, amino acids, and phosphate, sodium is reabsorbed and exchanged with chloride in the tubular region [29]. Potassium is freely filtered by the glomerulus and is reabsorbed in the proximal tubule and loop of Henle [31,32]. For the regulation of calcium excretion, most filtered calcium is reabsorbed at the renal tubules [33]. Once kidney damage occurs, the regulatory mechanism of excretion and reabsorption is dysfunctional, resulting in lower concentrations of electrolytes in the urine. A study in 2019 reported that low urinary potassium excretion is associated with CKD progression [34]. Another study also indicated that patients with lower eGFR had lower urine Na+ levels, especially those in CKD stage 5 [35]. These electrolytes are present in the ionic form in urine and therefore determined by UCond [22,27]. The lower the UCond, the lower the eGFR and the worse the kidney function. This relationship is supported by our results in that we found a significant correlation between UCond and eGFR (a correlation coefficient of 0.350 for males and 0.385 for females in the training set). These findings are consistent with the physiological trend and support that UCond eGFR is a physiologically reasonable predictor of eGFR. 

Previous studies reported that UCond is positively related to urine specific gravity (S.G.) [27], urine creatinine (UC) [23], and urine osmolality (UO) [27,36,37]. Urine S.G. is used to assess hydration status as regulated by the kidney through water reabsorption. In the study, we found a correlation coefficient of 0.628 between urine S.G. and UCond. Creatinine is a breakdown product of creatine phosphate in muscle [10]. It is used to determine the reference eGFR of CKD [8]. In a normal subject, the serum creatinine level is proportional to muscle mass. It is excreted to urine steadily as UC. We found that the correlation coefficient between UCond and UC was 0.446 for males and −0.113 for females. This gender difference may be due to a difference in muscle mass which is gender dependent. UO is useful in diagnosing renal disorders of hydration. In this study, we found a perfect correlation (coefficient of 1.000) between UCond and UO. Such a high correlation might be due to the UCond values being measured with Sysmex UF-5000, which generated UO [37]. 

The level of urine protein (UP) is widely recognized as a marker of CKD severity. It is also a predictor of future decline in glomerular filtration rate and an indicator of CKD risks [8,16]. The study used UP of <15.0 (mg/dL) for screening suspected early-stage CKD. However, the results showed that Sn, Sp, PPV, NPV, and accuracy for validation were 67.5%, 62.5%, 39.1%, 84.3%, and 63.8%, respectively, for males ages ≥ 45, while 75.0%, 57.1%, 35.7%, 87.8%, and 61.4%, respectively, for females ages ≥ 55. The lower sensitivity and accuracy will lead to losing many suspected cases for follow-up.

For quantitative analysis, either the albumin-to-creatinine ratio (ACR) or the protein-to-creatinine ratio (PCR) was recommended for initial albuminuria testing [19,21]. Once ACR or PCR was not available, urine dipsticks for albuminuria screening were used. A report concluded that the urine dipstick had poor Sn for ACR ≥ 30 mg/g [20]. In 2021, a meta-analysis study also pointed out that the urine dipstick presented high ranges of Sn in different categories for ACR. As a result, using the semiquantitative tool of urine dipstick to category ACR was already challenging, let alone screening for suspected early-stage CKD. However, the study lacked dipsticks for the comparisons. Nevertheless, in comparison to UP, proposed predicted models with UCond presented more accuracy and Sn for suspected early-stage CKD in the study.

Sn, Sp, PPV, and NPV are four standard measures to reflect the accuracy of a test [26]. In the study, participants with eGFR ≥ 60 (mL/min/1.73 m^2^) were regarded as having early-stage CKD for screening conditions. Based on the proposed formulae of P_male45 and P_female55, the Sn for males aged ≥ 45 and females aged ≥ 55 was 97.3% and 98.45, respectively, indicating a very high positive screening rate for suspected early-stage CKD. These highly sensitive screening tools mean that there are few false negative results, and thus fewer cases of early-stage CKD are missed. This may provide long time monitor for suspected early-stage CKD. Once the predicted value of eGFR is less than 60, it is recommended to go to the nephrology department for follow-up. In contrast, UP showed a low sensitivity of 67.5% and 75.0% for the same participants in the validation set. However, the proposed formulae presented Sp of only 17.5% and 30.0%. That meant a large proportion of false positives for the participants with eGFR < 60 (mL/min/1.73 m^2^) screening as early-stage CKD. Nevertheless, patients will experience some symptoms, such as high blood pressure, swelling in the legs, anemia, loss of appetite, or nausea [7,8]. Therefore, the physician can assess the kidney function to avoid the miss prediction. PPV indicated the probability of screening the disease after testing with a positive result was 76.7% and 81.6%, whereas NPV, the probability of not screening the disease after a negative test result was 70.0% and 85.7%, respectively. On the other hand, UP had 39.1% PPV and 84.3% NPV for males aged ≥45, while 35.7% PPV and 87.8% NPV for those aged ≥55. UP had very low proportions of positive predictions in the screening. In addition, our study showed that UCond was poorly correlated with UP and UG. Overall, P_male45 and P_femae55 represent likely a tool for screening suspected early-stage CKD in the risk population (i.e., DM) at an accuracy of 76.3% and 81.9%, respectively.

We used UCond in a spot urine sample to develop predictive formulae for eGFR. They may use to monitor the rate of change in renal function over time in future work. However, UCond and other laboratory data in the sample could vary due to underlying diseases, drugs, or diet. For example, high blood pressure or sleep apnea could elevate UP levels [38,39] but not UCond. This may bias the results. In addition, the measurement of Ucond does not indicate the levels of Na^+^, K^+^, Cl^−^, and Ca^++^ alone. Patients often take diuretics (i.e., Indapamide), angiotensin-converting enzyme inhibitors (i.e., Benazepril), or those with hyperkalemia could affect UCond [8]. Consequently, the association between eGFR and UCond is altered. Those related data were not reviewed for the retrospective study and were a limitation of the study.

## 5. Conclusions

Two predictive formulae have been developed for screening suspected early-stage CKD. Among the two, P_male45 was for males aged ≥45 with 76.3% accuracy and 97.3% sensitivity. The other P_female55 was for females aged ≥55 with 81.9% accuracy and 98.4% sensitivity. Since urine conductivity was poorly correlated with urine glucose and urine protein, our UCond-based predictive models for eGFR are likely less prone to interference by proteins present in the urine of patients with kidney injury or by glucose in diabetic patients. Our models have the desirable features of low cost and rapid detection. It may be put into practice for monitoring CKD in patients with progressive diabetes mellitus.

## Figures and Tables

**Table 1:** The characteristics of enrolled subjects.

	All (*n* = 600)	Training Set (*n* = 300)	Validation Set (*n* = 300)	*p*-Value
Age (years old)	61.9 ± 15.0	61.5 ± 14.6	62.3 ± 15.5	0.522
Sex				0.286
male/female (*n*, %)	332 (55.3)/268 (44.7)	159 (53.0)/141 (47.0)	173 (57.7)/127 (42.3)	
eGFR (mL/min/1.73 m^2^)	47.7 ± 26.6	46.6 ± 26.5	47.9 ± 26.7	0.917
≥90 (*n*, %)	39 (6.5)	19 (6.3)	20 (6.7)	
60–89 (*n*, %)	136 (22.7)	68 (22.7)	68 (22.7)	
30–59 (*n*, %)	268 (44.7)	134 (44.7)	134 (44.7)	
15–29 (*n*, %)	84 (14.0)	42 (14.0)	42 (14.0)	
<15 (*n*, %)	73 (12.2)	37 (12.3)	36 (12.0)	
Serum creatinine (mg/dL)	2.13 ± 1.93	2.14 ± 2.04	2.12 ± 1.82	0.908
Urine S.G.	1.014 ± 0.006	1.014 ± 0.006	1.014 ± 0.006	0.645
UO (mOsm/kg)	192.4 ± 85.4	193.9 ± 88.9	190.9 ± 81.8	0.670
Urine protein (mg/dL)	82.3 ± 195.3	81.3 ± 192.3	83.3 ± 198.0	0.900
<15 (mg/dL) (*n*, %)	305 (50.8)	150 (50.0)	155 (51.7)	
≥15 (mg/dL) (*n*, %)	295 (49.2)	150 (50.0)	145 (48.3)	
Urine protein <15 (mg/dL)	7.5 ± 3.2	7.5 ± 3.2	7.5 ± 3.2	0.934
Urine protein ≥ 15 ( mg/dL)	159.6 ± 256.7	155.1 ± 252.4	164.3 ± 261.9	0.759
Urine glucose (mg/dL) ^κ^	1282 ± 1519.6	1180.9 ± 1531.6	1366.2 ± 1520.4	0.572
Urine creatinine (mg/dL) ^λ^	86.1 ± 56.8	87.2 ± 59.6	84.8 ± 53.9	0.694
Urine Na^+^ (mEq/L)	70.2 ± 32.3	70.0 ± 32.6	70.8 ± 32.0	0.641
Urine K^+^ (mEq/L)	30.5 ± 18.6	31.3 ± 20.0	29.7 ± 17.2	0.289
Urine Cl^−^ (mEq/L)	65.7 ± 37.2	65.4 ± 37.8	66.0 ± 36.6	0.854
Urine Ca^++^ (mEq/L)	4.9 ± 5.5	5.1 ± 6.1	4.8 ± 4.9	0.516
UCond (mEq/L)	10.8 ± 4.6	10.9 ± 4.8	10.8 ± 4.4	0.698

^κ^: The number of urine glucose was 40 and 48 in the training set and validation set, respectively. ^λ^: The number for urine creatinine was 182 and 173, respectively. S.G.: Specific gravity; UO: urine osmolality; UCond: Urine conductivity; *p*-value was determined by independent *t*-test.

**Table 2:** The characteristics of enrolled subjects by sex difference.

	Male (*n* = 332)	Female (*n* = 268)	*p*-Value
Age (years old)	62.3 ± 14.5	61.4 ± 15.7	0.485
eGFR (mL/min/1.73 m^2^)	47.0 ± 23.5	48.7 ± 30.0	0.442
Serum creatinine (mg/dL)	2.24 ± 1.78	2.01 ± 2.11	0.144
Urine S.G.	1.014 ± 0.067	1.013 ± 0.006	0.013 *
UO (mOsm/kg)	194.2 ± 84.4	190.2 ± 86.7	0.563
Urine protein (mg/dL)	74.4 ± 152.3	92.1 ± 238.1	0.293
Urine glucose (mg/dL) ^κ^	1649.3 ± 1714.3	751.3 ± 983.5	0.003 *
Urine creatinine (mg/dL) ^λ^	95.3 ± 58.5	72.6 ± 51.6	0.000 *
UCond (mEq/L)	10.9 ± 4.6	10.7 ± 4.7	0.547

^κ^: The number of urine glucose was 53 and 36 in males and females, respectively. ^λ^: The numbers for urine creatinine were 211 and 144, respectively. *: *p* < 0.05. S.G.: Specific gravity; UO: urine osmolality; UCond: Urine conductivity; *p*-value was determined by independent *t*-test.

**Table 3:** The correlation analysis for eGFR and urine conductivity in the training set.

	eGFR			Urine Conductivity		
	All	Male	Female	All	Male	Female
Age	−0.288 *	−0.170 *	−0.385 *	0.043	0.096	−0.011
Urine S.G.	0.344 *	0.311 *	0.400 *	0.628 *	0.627 *	0.642 *
Urine osmolality	0.369 *	0.355 *	0.387 *	1.000 *	1.000 *	1.000 *
Urine protein	−0.244 *	−0.419 *	−0.155	−0.137 *	−0.186 *	−0.112
Urine glucose	0.278	0.416	0.225	0.038	0.040	−0.058
Urine creatinine	0.143	0.243 *	0.066	0.482 *	0.446 *	−0.113
Urine Na^+^	0.158 *	0.113	0.208 *	0.828 *	0.832 *	0.826 *
Urine K^+^	0.356 *	0.381 *	0.336 *	0.699 *	0.717 *	0.684 *
Urine Cl^−^	0.264 *	0.229 *	0.302 *	0.893 *	0.872 *	0.920 *
Urine Ca^++^	0.478 *	0.429 *	0.538 *	0.517 *	0.495 *	0.547 *
Urine conductivity	0.366 *	0.350 *	0.385 *	-	-	-

S.G.: Specific gravity. *: *p* < 0.05.

**Table 4:** The AUC analysis of urine conductivity for the fit-aged population.

eGFR = 90							
Age	≥45	≥50	≥55	≥60	≥65	≥70	All Ages
male	0.541	0.542	0.530	0.301	0.563	0.466	0.685
female	0.588	0.617	0.553	0.410	0.443	0.289	0.620
All	0.573	0.591	0.540	0.361	0.489	0.374	0.637
eGFR = 60							
Age	≥45	≥50	≥55	≥60	≥65	≥70	All ages
male	0.710	0.712	0.645	0.636	0.552	0.588	0.675
female	0.694	0.703	0.723	0.697	0.660	0.670	0.691
All	0.702	0.708	0.683	0.664	0.613	0.634	0.683

**Table 5:** Regression analysis for proposed predicted models.

	Nonstandardized Coefficients		*t*	*p*	*R* ^2^
	β	Standard Error			
Formula 2					0.152
Constant	25.541	4.465	5.721	0.000	
UCond	1.847	0.374	4.946	0.000	
Formula 3					0.137
Constant	26.686	4.676	5.707	0.000	
UCond	1.768	0.394	4.488	0.000	
Formula 4					0.245
Constant	69.563	16.419	4.237	0.000	
UCond	2.303	0.494	4.660	0.000	
Age	−0.752	0.222	−3.385	0.001	
Formula 5					0.238
Constant	78.160	19.077	4.097	0.000	
UCond	2.217	0.512	4.328	0.000	
Age	−0.854	0.259	−3.301	0.001	

UCond: urine conductivity.

**Table 6:** The validation of proposed predicted models and urine protein.

Subjects	Screening	Sn (%)	Sp (%)	Accuracy (%)	PPV (%)	NPV (%)
Male ≥ 45 (*n* = 152)	UCond (F2)	97.3	17.5	76.3	76.7	70.0
	Urine protein	67.5	62.5	63.8	39.1	84.3
Male ≥ 50 (*n* = 145)	UCond (F3)	99.1	7.90	75.2	75.2	75
	Urine protein	65.8	62.6	63.4	38.5	83.8
Female ≥ 50 (*n* = 93)	UCond (F4)	94.2	20.8	75.3	77.4	55.6
	Urine protein	79.2	53.6	60.2	37.3	88.1
Female ≥ 55 (*n* = 83)	UCond (F5)	98.4	30.0	81.9	81.6	85.7
	Urine protein	75.0	57.1	61.4	35.7	87.8

Sn: sensitivity; Sp: specificity; PPV: positive predictive value; NPV: negative predictive value; UCond: urine conductivity; F2–F5: Formula (2) to Formula (5).

## Data Availability

Not applicable.
